# Machine learning-based normal tissue complication probability model for predicting albumin-bilirubin (ALBI) grade increase in hepatocellular carcinoma patients

**DOI:** 10.1186/s13014-022-02138-8

**Published:** 2022-12-07

**Authors:** Anussara Prayongrat, Natchalee Srimaneekarn, Kanokporn Thonglert, Chonlakiet Khorprasert, Napapat Amornwichet, Petch Alisanant, Hiroki Shirato, Keiji Kobashi, Sira Sriswasdi

**Affiliations:** 1 Division of Radiation Oncology, Department of Radiology, Faculty of Medicine, Chulalongkorn University, Bangkok, Thailand; 2 Department of Anatomy, Faculty of Dentistry, Mahidol University, Bangkok, Thailand; 3 Graduate School of Biomedical Science and Engineering, Hokkaido University, Sapporo, Japan; 4 Global Station for Quantum Biomedical Science and Engineering, Global Institute for Cooperative Research and Education, Hokkaido University, Sapporo, Japan; 5 Department of Medical Physics, Hokkaido University Hospital, Sapporo, Japan; 6 Department of Radiation Medical Science and Engineering, Faculty of Medicine, Hokkaido University Graduate School of Medicine, Sapporo, Japan; 7 Research affairs, Faculty of Medicine, Chulalongkorn University, Bangkok, Thailand; 8 Center of Excellence in Computational Molecular Biology, Faculty of Medicine, Chulalongkorn University, Bangkok, Thailand; 9 Center for Artificial Intelligence in Medicine, Faculty of Medicine, Chulalongkorn University, Bangkok, Thailand

**Keywords:** Normal tissue complication probability model, Machine learning, Radiation-induced liver toxicity, Albumin-bilirubin grade, Hepatocellular carcinoma

## Abstract

**Purpose:**

The aim of this study was to develop a normal tissue complication probability model using a machine learning approach (ML-based NTCP) to predict the risk of radiation-induced liver disease in hepatocellular carcinoma (HCC) patients.

**Materials and methods:**

The study population included 201 HCC patients treated with radiotherapy. The patients’ medical records were retrospectively reviewed to obtain the clinical and radiotherapy data. Toxicity was defined by albumin-bilirubin (ALBI) grade increase. The normal liver dose-volume histogram was reduced to mean liver dose (MLD) based on the fraction size-adjusted equivalent uniform dose (2 Gy/fraction and α/β = 2). Three types of ML-based classification models were used, a penalized logistic regression (PLR), random forest (RF), and gradient-boosted tree (GBT) model. Model performance was compared using the area under the receiver operating characteristic curve (AUROC). Internal validation was performed by 5-fold cross validation and external validation was done in 44 new patients.

**Results:**

Liver toxicity occurred in 87 patients (43.1%). The best individual model was the GBT model using baseline liver function, liver volume, and MLD as inputs and the best overall model was an ensemble of the PLR and GBT models. An AUROC of 0.82 with a standard deviation of 0.06 was achieved for the internal validation. An AUROC of 0.78 with a standard deviation of 0.03 was achieved for the external validation. The behaviors of the best GBT model were also in good agreement with the domain knowledge on NTCP.

**Conclusion:**

We propose the methodology to develop an ML-based NTCP model to estimate the risk of ALBI grade increase.

**Supplementary Information:**

The online version contains supplementary material available at 10.1186/s13014-022-02138-8.

## Introduction

Liver cancer is the sixth most common cancer and the third leading cause of cancer deaths worldwide [1]. In Thailand, liver cancer is the most common cause of cancer death, and is considered a burden to the health care system. Liver cancer is treated using a multidisciplinary approach, including surgery, localized chemotherapy, systemic chemotherapy, radiotherapy (RT), and combined treatment [2]. RT is a promising treatment option serving as an efficient bridging therapy with conventional treatments, such as surgery and trans-arterial chemoembolization (TACE) and as a local ablative treatment for unresectable or medically inoperable disease [2–4]. A disadvantage of RT is that the curative dose might be reduced to spare normal liver tissue to avoid radiation-induced liver disease (RILD), which is a dose-limiting complication and can lead to deterioration of liver function followed by liver failure and death [5, 6]. Currently, there is no definitive treatment for RILD and its management is limited to symptomatic and supportive care.

Classic RILD is a dose-limiting toxicity after liver irradiation [7, 8], which rarely occurs in the modern RT era. More recent criteria used to evaluate the risk of liver toxicity are an increase in Child-Pugh (CP) score ≥ 2, and a change in albumin-bilirubin (ALBI) grade that is a more objective measure of liver function [9, 10]. The CP classification is a semi-quantitative assessment consisting of subjective (ascites, encephalopathy) and objective parameters (total bilirubin, albumin, and prothrombin time international normalized ratio). However, the subjective nature of some parameters can result in disparate CP scores between physicians. In contrast, the ALBI score is completely quantitative and designed as an outcome biomarker after RT. This score is currently considered as a reliable survival prediction model for hepatocellular carcinoma (HCC) patients and demonstrated similar or better performance compared with the conventional CP classification in various geographic regions [11, 12], particularly in HCC patients treated with RT [13–15].

Since the 1990’s, mathematical models have been proposed to quantitatively determine the correlation between RT dosimetric data and the toxicity of normal tissue complication probability (NTCP) models [16, 17]. Traditional NTCP models for predicting RILD using dose-volume data were based on a simplified characterization of the interaction between the radiation dose and normal tissue complication [10, 18–21]. However, because RILD is a highly complex and multifactorial toxicity, the biological heterogeneity between patients cannot be ignored. These factors suggest that novel data-driven approaches, such as regression-based statistical models and machine learning (ML) methods might be more appropriate [22]. A multivariable NTCP model integrating clinical and dose-volume factors was reported using conventional logistic regression with good performance [23]. However, the drawbacks were the assumption of linearity between the independent variables and outcome, the limitations of a complex relationship, and the requirement of little or no multicollinearity between variables.

The interest in artificial intelligence (AI) has grown within the radiation oncology community. As a subset of AI, ML algorithms are a valuable tool for exploring the relationship between a number of input features and the outcome despite low interpretability or the black-box effect and the restricted clinical application due to the model over/underfitting [22, 24]. The major advantages of an ML-based predictive model include an analysis of complex relationships, capability for dealing with a large amount of data or variables, and tuning the best set of parameters to obtain the high-performance prediction model [22, 25].

The purpose of this study was to develop NTCP models using a conventional statistical approach and advanced ML approach to predict the risk of toxicity in HCC patients that were treated with conformal RT techniques.

## Materials and methods

### Study population

We retrospectively collected the data of the HCC patients that were treated at between December 2006 and September 2018. The inclusion criteria were (i) Eastern Cooperative Oncology Group performance status of 0–2, (ii) available 3-dimensional dosimetric parameters, (iii) available follow-up data for tumor and liver toxicity, with ≥ 4 months of follow-up for nontoxicity patients. Patients with whole liver irradiation, progressive disease within 4 months, or an ALBI grade 3 at baseline were excluded.

The study was approved by the institutional review board (IRB no. 602/60) at King Chulalongkorn Memorial Hospital and Chulalongkorn University, Thailand.

### Radiation treatment

The patients were treated with conformal external beam RT techniques (three-dimension conformal radiotherapy (3D-CRT), intensity modulated radiotherapy (IMRT), or stereotactic body radiotherapy (SBRT)). The gross target volumes (GTVs) were contoured using contrast-enhanced computed tomography (CT) scans, and magnetic resonance image if available, with a 5- to 10-mm margin expanded to account for subclinical disease, setup uncertainty, and respiratory motion. For the traditional free-breathing technique, an additional internal target volume (ITV) was considered with a 5-mm margin from the GTV and another 5-mm was added for the planning target volume. Until January 2015, the deep expiratory breath-hold (DEBH) technique with Vision RT (Varian, Palo Alto, CA, USA) was used to track the patients’ surfaces during radiation, thus, ITV was neglected. The normal liver was contoured as the entire liver subtracted with GTV. Treatment delivery was performed using a 10- or 15-MV linear accelerator and the tumor was geometrically verified by daily cone-beam CT.

The patients’ dose-volume histograms (DVHs) were obtained from the Eclipse planning system version 8.6 (Varian Medical Systems, Palo Alto, CA, USA). The liver DVH was converted into a 2-Gy equivalent dose (EQD2) using a linear-quadratic-linear model with an α/β for normal liver of 2.0 Gy [18]. To simplify the entire DVH into a single measurement and simultaneously account for organ architecture, the generalized equivalent uniform dose (gEUD) was calculated from the differential DVH pairs (${v}_{i}$ and ${D}_{i}$ represent the volume and dose in the ${i}^{th}$ dose bin) using the following formula [26, 27] :

$$gEUD={ \left(\sum _{i}{v}_{i}{D}_{i}^{a}\right)}^{\raisebox{1ex}{$1$}\!\left/ \!\raisebox{-1ex}{$a$}\right.}$$

where $a$ is the volume effect parameter, with $a$ =1 representing the mean dose and $a$ >1 or $a$ <1 increasing the weight of the high and low dose regions, respectively [28]. The gEUD was calculated at various volume effect parameters (a = 0.01, 0.05, 0.1, 0.5, 1.0, and 2.0).

### Toxicity endpoint definition

The laboratory results were recorded at baseline prior to RT and at the worst result within 4 months after RT. The primary RILD endpoint in this study was an increase in ALBI grade by ≥ 1 grade (ALBI1+). Other RILD comprised an increased ≥2 CP score (CP2+), and grade ≥2 transaminitis according to the Common Toxicity Criteria of Adverse Events version 5.0 (CTCAE2+) that was diagnosed when the serum liver enzymes (aspartate aminotransferase, AST, and alanine aminotransferase, ALT) increased by ≥ 3-fold from the upper normal limit if the baseline was normal or by ≥3-fold from the baseline if the baseline was abnormal.

### Data preprocessing

The original input features comprised the patient’s age, sex, presence of portal vein thrombosis at the main trunk (main PVT), previous surgery, previous TACE, total radiation dose, number of fractions, radiation dose per fraction, radiation technique, tumor volume in cm^3^, tumor diameter in cm, normal liver volume in cm^3^, baseline liver function test (AST, ALT, alkaline phosphatase (ALP), total bilirubin, international normalized ratio), viral hepatitis B and C status, baseline CP score, baseline ALBI score and grade, and normal liver gEUD. Missing data were imputed using linear regression models trained on all input features.

A new feature based on baseline ALBI score was derived by calculating the difference between the baseline ALBI score and the next threshold for ALBI grading (at − 2.6 for the change from grade 1 to grade 2 and at -1.39 for the change from grade 2 to grade 3). This helped the models recognize whether a patient’s baseline ALBI score was close to the ALBI grade threshold and thus likely to result in an ALBI1 + outcome. This engineered feature, called the ALBI score until next grade, was also used to define a simple univariate model that the other models were compared to.

### NTCP model development

Three ML model families were evaluated for predicting ALBI1+: penalized logistic regression (PLR), random forest (RF), and gradient-boosted tree (GBT). A 5-fold cross-validation was performed to calculate the area under the receiver operating characteristic curve (AUROC) scores. Recursive feature eliminations were performed to identify the feature set that resulted in the highest AUROC was selected. When multiple dosimetric features were selected, we also evaluated alternative feature sets where only one dosimetric feature was included to keep the model simple and prevent overfitting. The details of feature selection process and hyperparameter tuning for each ML model was described in Supplement 1.

### NTCP model evaluations

Each model was evaluated on three aspects: internal validation AUROC (N = 201) from a 5-fold cross-validation, external validation AUROC (N = 44), and agreement between model behaviors with domain knowledge of how each input feature should impact the ALBI1 + outcome. In addition to evaluating individual models, the performance of a PLR and GBT model ensemble (combined model) was also considered because these two model families performed well on different partitions of the dataset.

To evaluate the behavior of each model, for PLR (linear model), the distribution of the model coefficients from the five models developed during the 5-fold cross-validation step was used. For RF and GBT, because these models are complex and non-linear, the impact of each input on the ALBI1 + outcome was estimated empirically by varying the value of each input feature in each patient from the minimum observed value to the maximum and calculating the changes in the model’s predictions compared with when the input was set at its mean value.

### Statistical analysis

The clinical and dosimetric parameters between patients with or without ALBI1 + were compared using the $\chi^{2}$test for categorical variables, and the independent samples *t*-test and the Mann-Whitney test for parametric and non-parametric variables, respectively. Statistical analyses were performed using Python version 3.8, XGBoost Python library [29], and SPSS (version 24.0, IBM Corp., Armonk, NY: IBM Corp.).

## Results

Two-hundred and one patients were assessable for ALBI1 + endpoint analysis and 199 patients for other endpoints. Their mean age was 61.7 year. Most were male (84.6%) and baseline CP class A (71.1%). Their mean tumor size and normal liver volume was 7.8 cm and 1,231.3 cm^3^, respectively. Median alpha-fetoprotein level was 101.3 ng/mL (interquartile range (IQR), 8.47–3090). Most patients received an RT dose of 30 Gy (IQR, 30–45 Gy) delivered in 10 fractions, and 20.9% of patients were treated with SBRT with median dose of 50 Gy (IQR, 33–50 Gy) delivered in a median of 5 fractions. The patient characteristics are presented in Table 1. Overall liver toxicity occurred in 87/201 patients (43.1%) with ALBI1+, 96/199 patients (48.2%) with CP2+, and 81/199 patients (40.7%) with CTCAE2+.

**Table 1:** Pretreatment characteristics of 201 hepatocellular carcinoma patients

	Total (n = 201)	No ALBI1+ (n = 114)	ALBI1+ (n = 87)	p-value
Age, mean (SD)	61.7 (11.7)	61.9 (11.3)	61.0 (12.3)	0.694
Sex, n (%)				0.309
Male	170 (84.6%)	99 (86.8%)	71 (81.6%)	
Female	31 (15.4%)	15 (13.2%)	16 (18.4%)	
Viral hepatitis carrier, n (%)				
Viral hepatitis B	99 (49.3%)	57 (50.0%)	42 (48.2%)	0.809
Viral hepatitis C	38 (18.9%)	22 (19.3%)	16 (18.3%)	0.871
Presence of main portal vein thrombosis, n (%)	76 (37.8%)	41 (35.9%)	35 (40.2%)	0.537
Other treatments, n (%)				
Surgery	25 (12.4%)	18 (15.8%)	7 (8.0%)	0.099
TACE	127 (63.2%)	77 (67.5%)	50 (57.5%)	0.142
Tumor size (cm), mean (SD)	7.8 (4.1)	7.5 (3.9)	8.3 (4.3)	0.185
Baseline liver function test, mean (SD)				
AST (U/L)	68.6 (41.8)	62.8 (38.2)	76.1 (45.1)	0.029
ALT (U/L)	44.7 (28.8)	43.2 (27.7)	46.7 (30.2)	0.392
Total bilirubin (mg/dL)	1.4 (0.7)	1.1 (0.6)	1.3 (0.8)	0.043
Baseline ALBI score, mean (SD)	-2.28 (0.43)	-2.24 (0.37)	-2.19 (0.50)	0.377
Baseline ALBI, n (%)				< 0.001
Grade 1	42 (20.9%)	12 (10.5%)	30 (34.5%)	
Grade 2	159 (79.1%)	102 (89.5%)	57 (65.5%)	
Radiotherapy techniques, n (%)				
3D-CRT	66 (32.8%)	35 (30.7%)	31 (35.6%)	0.461
IMRT, VMAT	93 (46.3%)	55 (48.2%)	38 (43.7%)	0.520
SBRT	42 (20.9%)	24 (21.1%)	18 (20.7%)	0.950
Dose-volume data, median (IQR)				
Total dose (Gy)	33 (30–45)	40 (30–45)	30 (30–45)	0.097
Number of fractions	10 (10–10)	10 (10–14)	10 (8–10)	0.120
Gross tumor volume (cm^3^)	177.8 (742.8)	155.0 (540.8)	264.4 (855.3)	0.100
Dose-volume data, mean (SD)				
Normal liver volume (cm^3^)	1231.3 (455.6)	1222.0 (403.6)	1243.4 (518.1)	0.742
Normal liver gEUD at a = 1.0 or mean liver dose (Gy)	17.6 (8.0)	16.8 (7.2)	18.7 (8.8)	0.088

SD = standard deviation; IQR = interquartile range

ALBI = albumin-bilirubin score; TACE = transarterial chemoembolization; AST = aspartate aminotransferase; ALT = alanine aminotransferase; 3D-CRT = three-dimensional conformal radiotherapy; IMRT = intensity modulated radiotherapy; VMAT = volumetric modulated arc therapy; SBRT = stereotactic body radiotherapy; gEUD = generalized equivalent uniform dose

### ALBI1 + predictor behavior

**Fig. 1:**
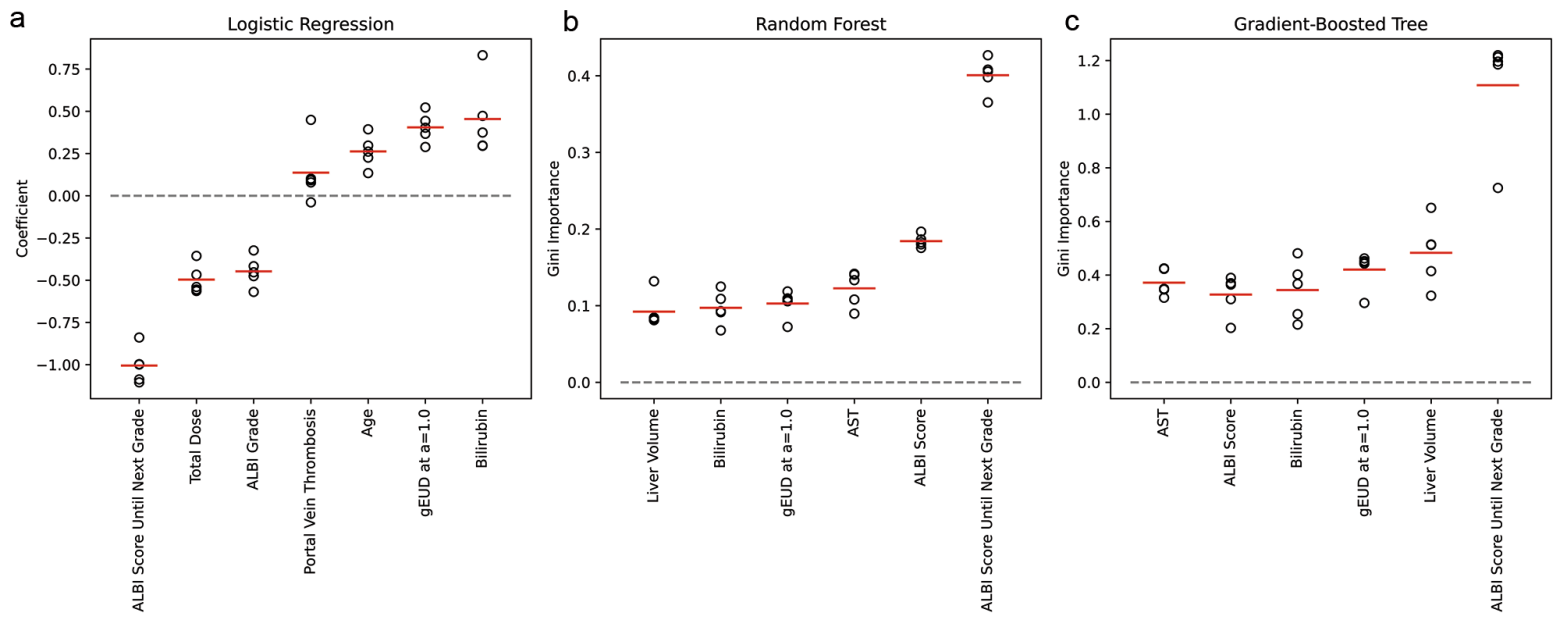
Importance of each input feature for the best model. (a) Logistic regression model. Scatter plots of the coefficient values from the five logistic regression models trained using different partitions of the 5-fold cross-validation. Features with higher absolute magnitudes are considered more important to the prediction. Red bars indicate the average values. (b) Random forest model. Scatter plots of the Gini importance values from the five random forest models trained using different partitions of the 5-fold cross-validation. Features with higher Gini importance score are considered more important to the prediction. Red bars indicate the average values. (c) Gradient-boosted tree model. Scatter plots of the Gini importance values from the five gradient-boosted tree models trained using different partitions of the 5-fold cross-validation. Red bars indicate the average values

**Fig. 2:**
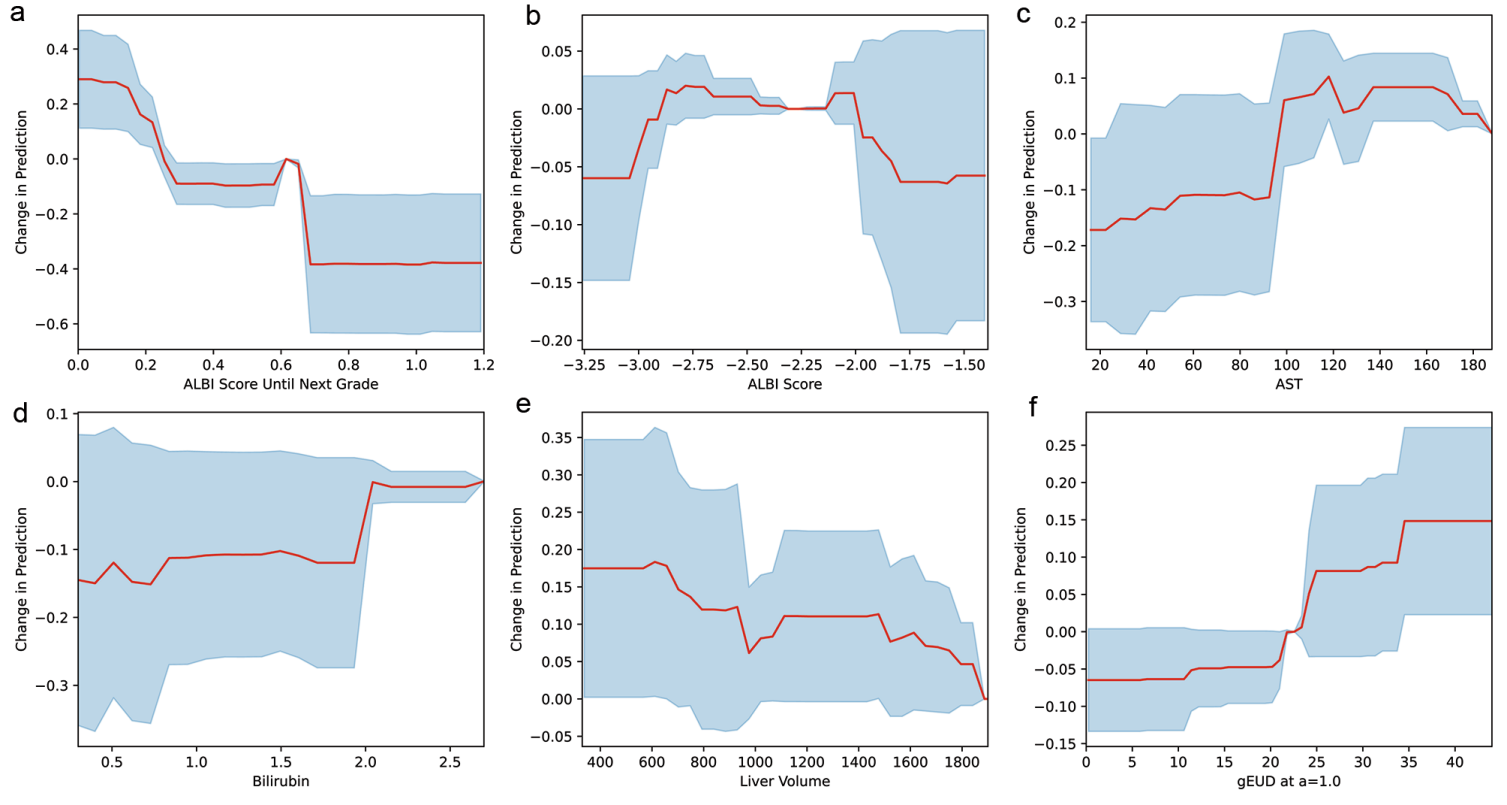
Behaviors of the best gradient-boosted tree model. The impact of each input feature on the prediction was estimated empirically by altering the feature value and recording the corresponding change in the model’s output. Red trend lines show the average relative change in prediction. Blue shaded areas indicate the plus/minus one standard deviation range. (a) Impact of the ALBI score until next grade on the prediction. (b) Impact of the baseline ALBI score on the prediction. (c) Impact of the baseline AST on the prediction. (d) Impact of the baseline total bilirubin on the prediction. (e) Impact of normal liver volume on the prediction. (f) Impact of the gEUD calculated at a = 1.0, or mean liver dose, on the prediction

The behaviors of the PLR model were investigated through its coefficients (Fig. 1). ALBI score until the next grade was the most important feature followed by total dose, total bilirubin, baseline ALBI grade, and gEUD (Fig. 1a). The gEUD at a = 1.0, or mean liver dose (MLD), was the most predictive among various volume effect parameters and adopted in the model analysis (Supplementary Table 1). Total bilirubin and MLD contributed positively to the likelihood of ALBI1+, and ALBI score until the next grade contributed negatively to the likelihood of ALBI1+. There were some discrepancies, such as the negative coefficients of the total dose and baseline ALBI grade, which reflected the limitation of the linear model. For the RF and GBT models, the importance of the ALBI score until the next grade was twice that of the other features (Fig. 1b, 1c). The model behaviors, as estimated by altering the input feature values and measuring the changes in prediction outputs, closely followed expectations from domain knowledge (Fig. 2 for the GBT model and Supplementary Fig. 1 for the RF model). The closer the patient’s baseline ALBI score was to the next grade threshold, the higher the likelihood of ALBI1+. Poorer liver function (higher bilirubin or AST) and radiation dose conferred a higher risk of toxicity, while higher normal liver volume resulted in lower risk. The change in ALBI prediction was abrupt at MLD = 21 Gy regardless of baseline ALBI grade and liver volume (Supplementary Fig. 2).

### Development of machine learning models for predicting ALBI1 + outcome

The use of dose feature or baseline ALBI score yielded a considerably lower AUROC of 0.5341–0.6198 (Supplementary Table 1). To improve model performance, several clinical and treatment factors were integrated into the model. When clinical factors were considered, the model AUROC was improved to 0.7657 for the PLR model, 0.7904 for the RF model, and 0.7911 for the GBT model (Supplementary Table 2). Noting that, ALBI score until next grade was included in all models. The inclusion of treatment features into the model further improved the AUROC to 0.7864 for the PLR model, 0.8076 for the RF model, and 0.8166 for the GBT model (Table 2).

**Table 2:** Internal validation of each NTCP model for predicting ALBI1+.

	AUROC (mean ± SD)	Input Features
ALBI score until next grade	0.7657 ± 0.0738	ALBI score until next grade
Logistic regression (PLR)	0.7864 ± 0.0848	Age, ALBI score until next grade, baseline ALBI grade, total bilirubin, portal vein thrombosis, total dose, and MLD
Random forest (RF)	0.8076 ± 0.0583	AST, ALBI score until next grade, baseline ALBI score, total bilirubin, normal liver volume, and MLD
Gradient-boosted tree (GBT)	0.8166 ± 0.0709	AST, ALBI score until next grade, baseline ALBI score, total bilirubin, normal liver volume, and MLD
Ensemble model (PLR + GBT)	0.8214 ± 0.0605	PLR and GBT Inputs

Abbreviation: PLR = Penalized logistic regression; RF = Random forest; GBT = Gradient-boosted tree; AUROC = area under the receiver operating characteristic curve; SD = standard deviation; ALBI = albumin-bilirubin score; AST = aspartate aminotransferase; MLD = mean liver dose (gEUD at a = 1.0)

**Fig. 3:**
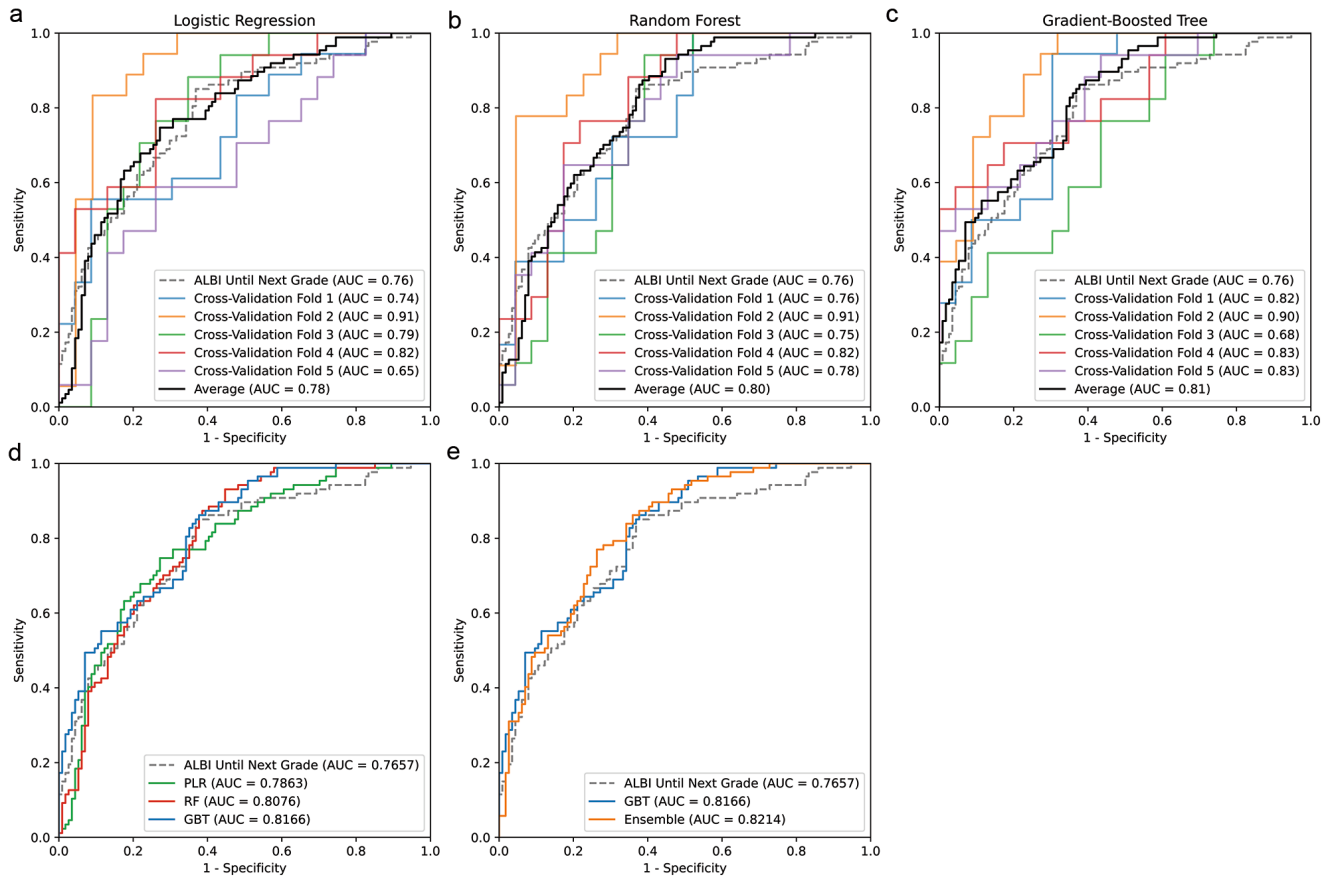
Internal 5-fold cross-validation results of NTCP models. A simple model using only baseline ALBI score until next grade as input is shown as a reference (gray dashed line). The AUROC scores are indicated in the legend for each model. (a) ROC curves for the best logistic regression models from each cross-validation fold (colored solid lines) and the average (black solid line). (b) ROC curves for the best random forest models. (c) ROC curves for the best gradient-boosted tree models. (d) Comparison of the average ROC curves for the best logistic regression (PLR, green), random forest (RF, red), and gradient-boosted tree (GBT, blue) models. (e) Comparison of the average ROC curves for the best gradient-boosted tree models (GBT, blue) and the ensemble of the best logistic regression models and the best gradient-boosted tree models (Ensemble, orange)

The 5-fold cross-validation results revealed that the different model families excelled on different partitions of the dataset (Fig. 3a, 3b, 3c). Although the PLR model performed well on Fold 3 with an AUROC of 0.79, the GBT model, which achieved the highest average AUROC, yielded an AUROC of only 0.68 on the same data. In contrast, the GBT model achieved an AUROC of 0.83 on Fold 5, while the PLR model achieved an AUROC of only 0.65. Furthermore, the PLR and GBT models also performed well on different regions of the ROC curve, with the GBT model being the best in the high (> 0.8) and low (< 0.6) specificity regions and the PLR model being the best in the intermediate specificity region (Fig. 3d). These observations strongly suggested that an ensemble model constructed by averaging the outputs from the best PLR model and the best GBT model should outperform each individual model. Indeed, by combining the two model families, the ensemble model achieved a higher AUROC of 0.8214 (Fig. 3e).

**Fig. 4:**
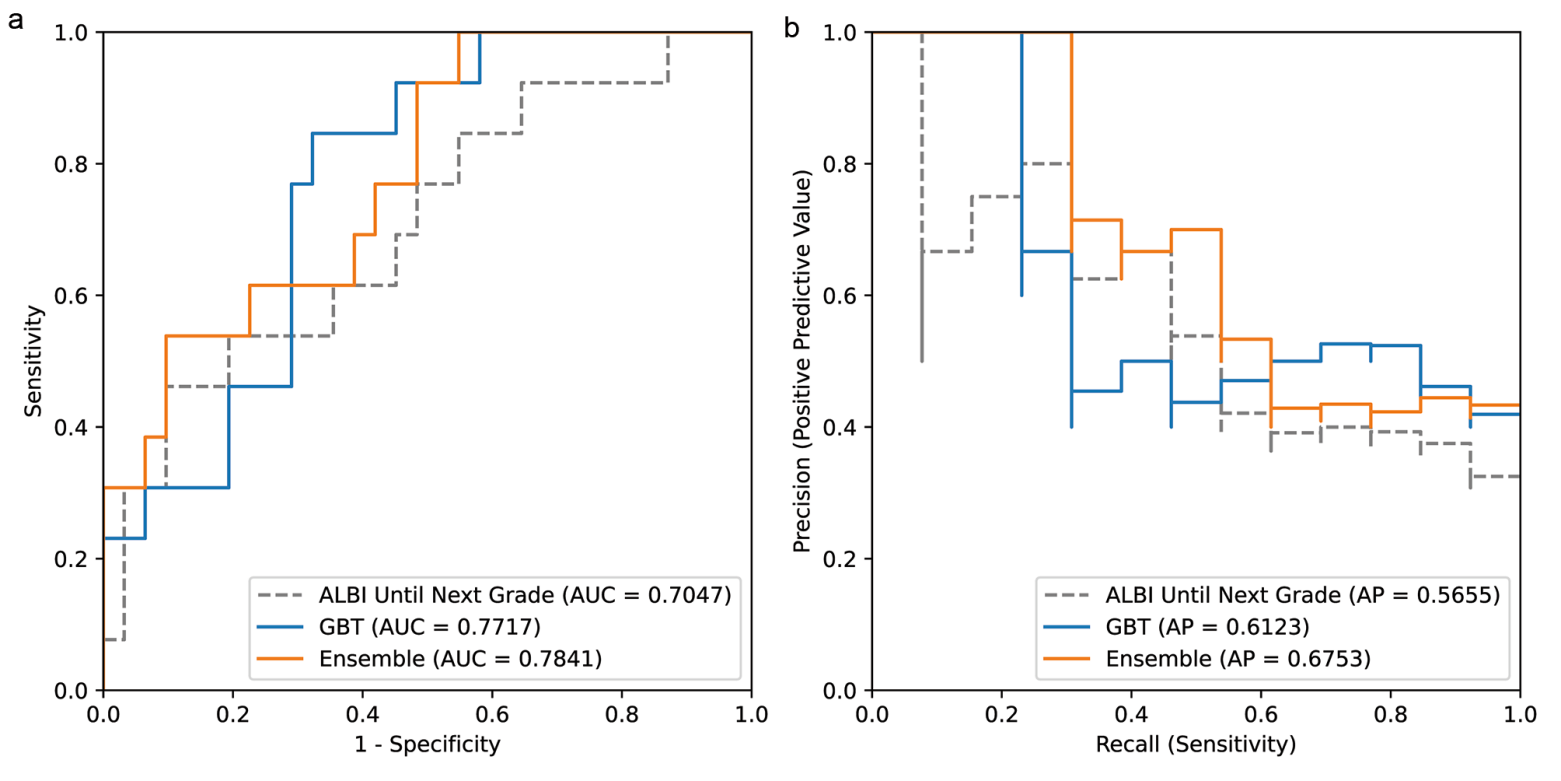
External validation (N = 44) performances of the best gradient-boosted tree model and ensemble model. (a) ROC curves for the simple model using only the baseline ALBI score as input (ALBI only, gray dashed line), the best gradient-boosted tree model (GBT, solid blue line), and the ensemble of logistic regression and gradient-boosted tree model (Ensemble, solid orange line). The AUROC is indicated for each model. (b) Precision-recall curves for the models shown in A). Average precision (AP) was indicated for each model

Using an external validation dataset (N = 44), the ensemble model also outperformed the simple univariate model and the best GBT model in AUROC and average precision metrics (AP), achieving an AUROC of 0.7841 and an AP of 0.6753 (Fig. 4a and 4b). Although the difference between the two models was small, the ensemble model was more desirable for NTCP predictions because it performed better than the GBT model in the high precision and high specificity regions.

### Predictability of other outcomes

In addition to ALBI1+, the predictability of other raw outcome scores and categorical outcomes were also assessed using the PLR and logistic regression models. The results demonstrated that the ALBI and CP scores were somewhat predictable with mean absolute errors of 0.4387 and 1.2190, respectively, while the worst AST, ALT, and total bilirubin scores were not (Supplementary Fig. 3). For the categorical outcomes, CTCAE2 + was quite difficult to predict, with an AUROC of 0.6878 and an AP of 0.4671, while the performance for CP2 + was more promising with an AUROC of 0.7261 and an AP of 0.7125 (Supplementary Table 3). However, the best CTCAE2 + and CP2 + models required 16 and 14 input features, respectively, including multiple gEUD features at various *a*’s, which made the models difficult to interpret and at risk of overfitting.

## Discussion

We introduced the ML-based prediction model to identify the HCC patients at risk of developing ALBI1 + using combined logistic regression and tree-based approaches. The model applied clinical and dosimetric parameters (age, main PVT, baseline AST and total bilirubin, baseline ALBI score and score until next grade, normal liver volume, and MLD). Using a combination of these features, the model performance was considerably improved compared with the dosimetric or baseline ALBI model.

Feature importance was in line with other studies. Our group reported a multivariable NTCP model using conventional statistical analysis to predict the risk of RILD in primary liver cancer patients, including HCC and cholangiocarcinoma [23]. The significant predictors consisted of tumor type, CP classification, hepatitis status, and normal liver dosimetry. Despite a high AUROC (0.79), the model lacked external validation and the complex interaction among variables was not optimally evaluated. To further enhance model reproducibility and utility, the current study focused on a specific tumor type (HCC only), more objective outcome (ALBI1+), and non-linear correlation between variables. The good discriminative ability of our model on internal and external validation (AUROC of 0.8214 and 0.7841, respectively) suggests that it is a potentially effective clinical decision support tool. In addition to estimating the toxicity risk from each treatment plan, the NTCP model was useful for selecting the radiation technique/ modality, the so-called NTCP model-based approach [30].

Each ML model family has distinct advantages and disadvantages. Although RF and GBT can capture higher-order feature interactions, they are prone to overfitting by exploiting spurious associations between input features and outcome and their behaviors can be difficult to interpret. In contrast, the simplistic nature of linear models makes them highly explainable but limits their ability to capture feature interactions. This tradeoff between complex and simple model families was seen in the complementary ALBI1 + AUROC of the GBT and PLR models on folds 3 and 5. The GBT model likely overfitted to the training dataset on fold 3, while the PLR model was unable to fit the training data on fold 5. By combining multiple models into an ensemble, the non-systematic errors produced by the models can cancel each other out to produce more accurate predictions. In this study, the ensemble of the GBT and PLR models demonstrated the best performance on both internal and external validations.

In addition to high performance, an ML-based model for medical applications should also behave in line with domain knowledge. Higher radiation doses or poorer liver function test scores resulted in higher likelihoods of ALBI1 + outcome. Notably, our GBT models were constrained to make the dosimetric features contribute positively to the toxicity risk. Although this constraint was not absolutely necessary, because unconstrained tree-based models can also somewhat recapitulate this behavior, using this constraint significantly reduced the variance of the model behavior and helped the model learn the negative association between normal liver volume and ALBI1 + outcome.

Pursley et al. developed an NTCP model to predict CP2 + and ALBI1 + in 108 HCC patients treated with SBRT and proton therapy and demonstrated the significance of low-dose bath to the liver (V5 and V10) [10]. However, MLD was suggested to be the most predictive in our study which was represented by best fit of gEUD at a = 1.0. This finding corresponded with the subset analysis in Pursley et al. that excluded proton patients where the fit lost its sensitivity to low-dose bath and the a-value best fit range included 1.0. This might be due to majority of patients in our cohort received less conformal RT techniques. In addition, our study found a higher incidence of ALBI1+ (43.1% vs. 34.4% in [10]) and a clearer dose-response relationship for ALBI1+. Possible explanations for these findings might be the poorer prognosis in our patient population and Pursley et al. including viral hepatitis 68% (B 49%, C 19%) versus 46% (B 9%, C37%), median baseline ALBI score − 2.3 (-3.3 to -1.4) versus − 2.6 (-3.8 to -0.9), and baseline ALBI grade 1 21% versus 45% respectively.

The strengths of this study include the diverse range of available RT techniques (3D-CRT, IMRT/VMAT, and SBRT) and the use of real-world data, ensuring a large variation in the dose distribution across the cohort. The use of gEUD, which were adjusted for fraction size to account for the heterogeneous RT regimens, increases the generalizability of the model. Furthermore, the presented ML algorithms behave in concordance with the knowledge of complex interactions between input features and the outcome, thus potentially improving the interpretability and generalizability of the model. Other advantages of our model include the easily obtained model parameters and its accessibility and reproducibility across other cancer types and other institutions (Python codes are available from the corresponding authors upon request).

The limitations of this study were its retrospective nature and relatively small size of the cohort. The heterogeneity of disease characteristics and a lack of data in the high dose region affected the predictive power of the model [31]. We reduced the potential biases by recruiting all patients in the study time-period to minimize selection bias, using multivariate analysis to adjust for confounding factors, and using the ALBI score as the primary endpoint to reduce the bias introduced by subjective clinical judgement. Although the main advantages of the ALBI score are its objective nature, simple use, and routine laboratory investigations, the ALBI1 + score is not typically applied in routine practice and still requires meticulous investigation in a larger population. Another concern of this study was that our patients had a substantial prevalence of viral hepatitis, poor baseline liver function, and relatively more advanced disease. Thus, the results may not be generalizable and the model requires external validation prior to its clinical use, especially in a population with different clinical presentations.

An updated review of the technological innovation of magnetic resonance linear accelerators has demonstrated promising clinical advantages in liver malignancies [32]. The magnetic resonance-guided RT (MRgRT) workflow allows accurate tumor and organs-at-risk segmentation, effective motion management, and online treatment plan adaptation. Advance RT strategies such as MRgRT and proton beam therapy enable the approach to treat the liver tumors with curative radiation dose while reducing the irradiated normal liver tissue. Therefore, liver cancer patients treated with MRgRT is a unique entity that warrants further study. In addition, other possible predictive markers, including platelet-albumin-bilirubin (PALBI) score, radiogenomic data, and biological markers, such as circulating inflammatory proteins should be integrated into the NTCP model to improve its predictive ability [13, 25, 33–36 ].

Unlike the statistical models [23, 37], our ensemble model could not readily provide the absolute NTCP value without an additional calibration on a larger external dataset. In other words, the predicted ALBI1 + risk score did not numerically reflect the toxicity risk, thus, limited the compatibility with the NTCP model-based approach for treatment selection. Further steps are needed before clinically applying the model including model calibration, model uncertainty assessment, and optimal cut-off value determination.

## Conclusion

This study illustrated the methodology of an ML approach to develop an NTCP model integrating clinical and dosimetric parameters to predict liver toxicity in HCC patients. The process should be reproducible and applicable to other types of cancers and toxicity outcomes.

## Electronic supplementary material

Below is the link to the electronic supplementary material.

Supplementary Materials.
Supplement 1. Feature selection process and hyperparameter tuning for each ML model.
Supplementary Table 1. Performance of ALBI1+ prediction using dose or raw ALBI score.
Supplementary Table 2. Input features and performance of models using clinical and dose features.
Supplementary Table 3. Predictability of the categorical outcomes using penalized logistic regression.
Supplementary Figure 1. Behaviors of the best random forest model.
Supplementary Figure 2. Impact of mean liver dose (gEUD at a=1.0) on patient subpopulations.
Supplementary Figure 3. Predictability of the raw outcome scores using penalized linear regression.

## Data Availability

The datasets analyzed for this study are available on request from the corresponding authors. Data and Python codes are available from the corresponding authors upon request.

## List of abbreviations

3D-CRT: three-dimension conformal radiotherapy.
AI: artificial intelligence.
ALBI: albumin-bilirubin.
ALP: alkaline phosphatase.
ALT: alanine aminotransferase.
AP: average precision metrics.
AST: aspartate aminotransferase.
AUROC: area under the receiver operating characteristic curve.
CP: Child-Pugh.
CT: computed tomography.
CTCAE: common toxicity criteria of adverse events.
DEBH: deep expiratory breath-hold.
DVH: dose-volume histogram.
EQD2: 2-Gy equivalent dose.
GBT: gradient-boosted tree.
gEUD: generalized equivalent uniform dose.
GTV: gross target volumes.
HCC: hepatocellular carcinoma.
IMRT: intensity modulated radiotherapy.
IQR: interquartile range.
ITV: internal target volume.
ML: machine learning.
MRgRT: magnetic resonance-guided radiotherapy.
NTCP: normal tissue complication probability.
PALBI: platelet-albumin-bilirubin.
PLR: penalized logistic regression.
PVT: portal vein thrombosis.
RF: random forest.
RILD: radiation-induced liver disease.
RT: radiotherapy.
SBRT: stereotactic body radiotherapy.
TACE: trans-arterial chemoembolization.
