# Correction: Machine learning-based normal tissue complication probability model for predicting albumin-bilirubin (ALBI) grade increase in hepatocellular carcinoma patients

**DOI:** 10.1186/s13014-023-02212-9

**Published:** 2023-03-15

**Authors:** Anussara Prayongrat, Natchalee Srimaneekarn, Kanokporn Thonglert, Chonlakiet Khorprasert, Napapat Amornwichet, Petch Alisanant, Hiroki Shirato, Keiji Kobashi, Sira Sriswasdi

**Affiliations:** 1 Division of Radiation Oncology, Department of Radiology, Faculty of Medicine, Chulalongkorn University, Bangkok, Thailand; 2 Department of Anatomy, Faculty of Dentistry, Mahidol University, Bangkok, Thailand; 3 Graduate School of Biomedical Science and Engineering, Hokkaido University, Sapporo, Japan; 4 Global Station for Quantum Biomedical Science and Engineering, Global Institute for Cooperative Research and Education, Hokkaido University, Sapporo, Japan; 5 Department of Medical Physics, Hokkaido University Hospital, Sapporo, Japan; 6 Department of Radiation Medical Science and Engineering, Faculty of Medicine, Hokkaido University Graduate School of Medicine, Sapporo, Japan; 7 Research Affairs, Faculty of Medicine, Chulalongkorn University, Bangkok, Thailand; 8 Center of Excellence in Computational Molecular Biology, Faculty of Medicine, Chulalongkorn University, Bangkok, Thailand; 9 Center for Artificial Intelligence in Medicine, Faculty of Medicine, Chulalongkorn University, Bangkok, Thailand

**Correction: Radiation Oncology (2022) 17:202** 10.1186/s13014-022-02138-8

After publication of this article [1], the authors reported that Sira Sriswasdi should have been denoted as co-corresponding author.

The original article [1] has been corrected.
