# Supplementary material for: Machine learning-based normal tissue complication probability model for predicting albumin-bilirubin (ALBI) grade increase in hepatocellular carcinoma patients
Source: Radiat Oncol. 2022 Dec 7;17:202. doi: 10.1186/s13014-022-02138-8 (PMC9730671; doi:10.1186/s13014-022-02138-8)
Supplement: Supplementary file 1 — Supplementary Materials. Supplement 1. Feature selection process and hyperparameter tuning for each ML model. Supplementary Table 1. Performance of ALBI1+ prediction using dose or raw ALBI score. Supplementary Table 2. Input features and performance of models using clinical and dose features. Supplementary Table 3. Predictability of the categorical outcomes using penalized logistic regression. Supplementary Figure 1. Behaviors of the best random forest model. Supplementary Figure 2. Impact of mean liver dose (gEUD at a=1.0) on patient subpopulations. Supplementary Figure 3. Predictability of the raw outcome scores using penalized linear regression. [file 13014_2022_2138_MOESM1_ESM.docx]

Supplementary Material

**Supplement 1.**

**Feature selection process and hyperparameter tuning for each ML model**

During the feature selection process, default hyperparameters were used for each model. Specifically, the PLR model with a L2-regularization strength of 1, the RF model with 100 decision trees, maximum depth of 3, and minimum leaf size of 9, and the GBT model with a maximum depth of 10, learning rate of 0.01, minimum child weight of 0.5, and L2-regularization strength of 10 were used. Each GBT model was trained for a maximum of 1000 iterations and could terminate early if validation performance stopped improving over 10 iterations (early stopping).

Once the feature sets that achieved the highest AUROC were identified for each model family, hyperparameter tuning was performed. For PLR, L1- and L2-regularization with strengths ranging from 0.01–10000 were evaluated. For RF, the number of trees ranging from 100–500, maximum depth ranging from 3 to unlimited, and minimum leaf size ranging from 1–9 were tested. For GBT, the maximum depth ranging from 3 to unlimited and minimum child weight ranging from 0.5–1.0 were tested. Furthermore, because the XGBoost Python library [Chen T, Guestrin C. XGBoost: A Scalable Tree Boosting System. Proceedings of the 22nd ACM SIGKDD International Conference on Knowledge Discovery and Data Mining. San Francisco, California, USA: New York, NY, USA: ACM; 2016. p. 785-94.] that was used to train the GBT model allowed monotonic constraints to be enforced on each input feature, the dosimetric features were constrained to contribute positively to the prediction. Thus, the GBT models were forced to predict higher likelihoods of ALBI1+ outcome if the input radiation dose increased. Other hyperparameters were not tuned because our preliminary analysis revealed that they had little impact on model performance. The combination of hyperparameters that yielded the highest AUROC from 5-fold cross-validation was selected for each model family.

**Supplementary Table 1. Performance of ALBI1+ prediction using dose or raw ALBI score**

| **Feature** | **Baseline ALBI score** | **Baseline ALBI grade** | **gEUD at a = 0.01** | **gEUD at a = 0.05** | **gEUD at a = 0.1** | **gEUD at a = 0.5** | **gEUD at a = 1.0** | **gEUD at a = 2.0** |
| --- | --- | --- | --- | --- | --- | --- | --- | --- |
| **AUROC** | 0.5341 | 0.6198 | 0.5606 | 0.5615 | 0.5620 | 0.5720 | 0.5738 | 0.5443 |
| **Average Precision** | 0.5531 | 0.5299 | 0.5172 | 0.5178 | 0.5212 | 0.5376 | 0.5337 | 0.4890 |

Abbreviation: AUROC= area under the receiver operating characteristic curve; ALBI=albumin-bilirubin score; gEUD=generalized equivalent uniform dose; a=volume effect parameter

**Supplementary Table 2.**

| **Model** | **AUROC** | **Input features** |
| --- | --- | --- |
| **Clinical features only** | | |
| PLR | 0.7657 | ALBI score until next grade |
| RF | 0.7904 | Baseline ALBI score, ALBI score until next grade, AST, GTV, liver volume, bilirubin |
| GBT | 0.7911 | ALBI score until next grade, AST, GTV, liver volume, bilirubin |
| **Clinical and treatment features** | | |
| ALBI score until next grade | 0.7657 | ALBI score until next grade |
| PLR | 0.7864 | Age, baseline ALBI grade, baseline ALBI score, bilirubin, portal vein thrombosis, total dose, gEUD at a=1.0 |
| RF | 0.8067 | AST, baseline ALBI score, bilirubin, liver volume, gEUD at a=1.0 |
| GBT | 0.8214 | AST, baseline ALBI score, bilirubin, liver volume, gEUD at a=1.0 |

Abbreviation: PLR= Penalized logistic regression; RF= Random forest; GBT= Gradient-boosted tree; AUROC= area under the receiver operating characteristic curve; ALBI=albumin-bilirubin score; AST= aspartate aminotransferase; GTV=gross tumor volume; gEUD=generalized equivalent uniform dose; a=volume effect parameter

**Supplementary Table 3. Predictability of the categorical outcomes using penalized logistic regression**

| **Outcome** | **AUROC** | **Average Precision** |
| --- | --- | --- |
| **ALBI1+** | 0.7864 | 0.7252 |
| **CP2+** | 0.7261 | 0.7125 |
| **CTCAE2+** | 0.6878 | 0.4671 |

Abbreviation: AUROC= area under the receiver operating characteristic curve; ALBI1+ = albumin-bilirubin grade increase by ≥1 grade; CP2+ = Child-Pugh (CP) score ≥2; CTCAE2+ = grade ≥2 transaminitis according to the Common Toxicity Criteria of Adverse Events version 5.0


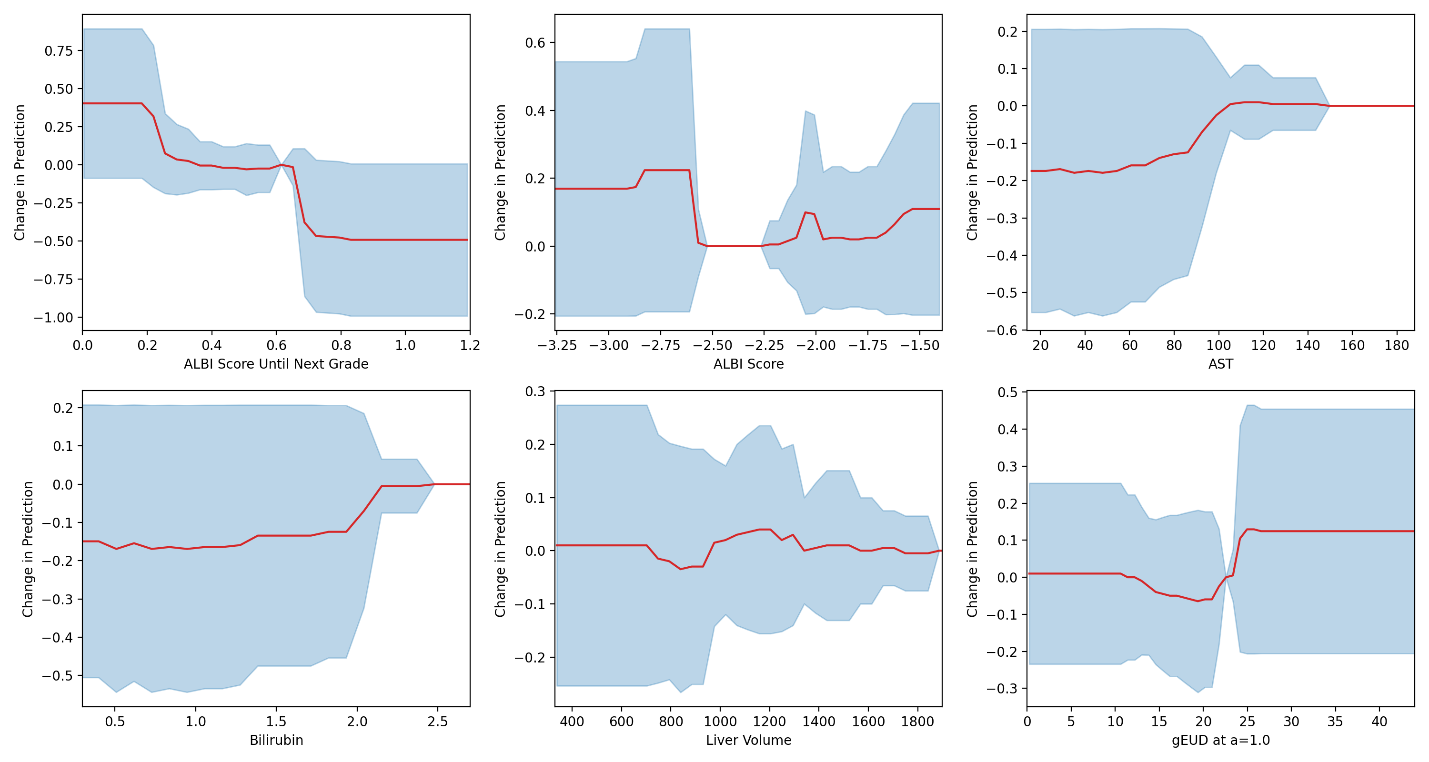


**Supplementary Figure 1. Behaviors of the best random forest model.** The impact of each input feature on the prediction was estimated empirically by altering the input feature value and recording the corresponding change in model’s output. Red trend lines show the average relative change in prediction. Blue shaded areas indicate the plus/minus one standard deviation range. A) Impact of the ALBI score until next grade on the prediction. B) Impact of the baseline ALBI score on the prediction. C) Impact of the baseline AST on the prediction. D) Impact of the baseline bilirubin on the prediction. E) Impact of the normal liver volume on the prediction. F) Impact of the gEUD calculated at a = 1.0 on the prediction.


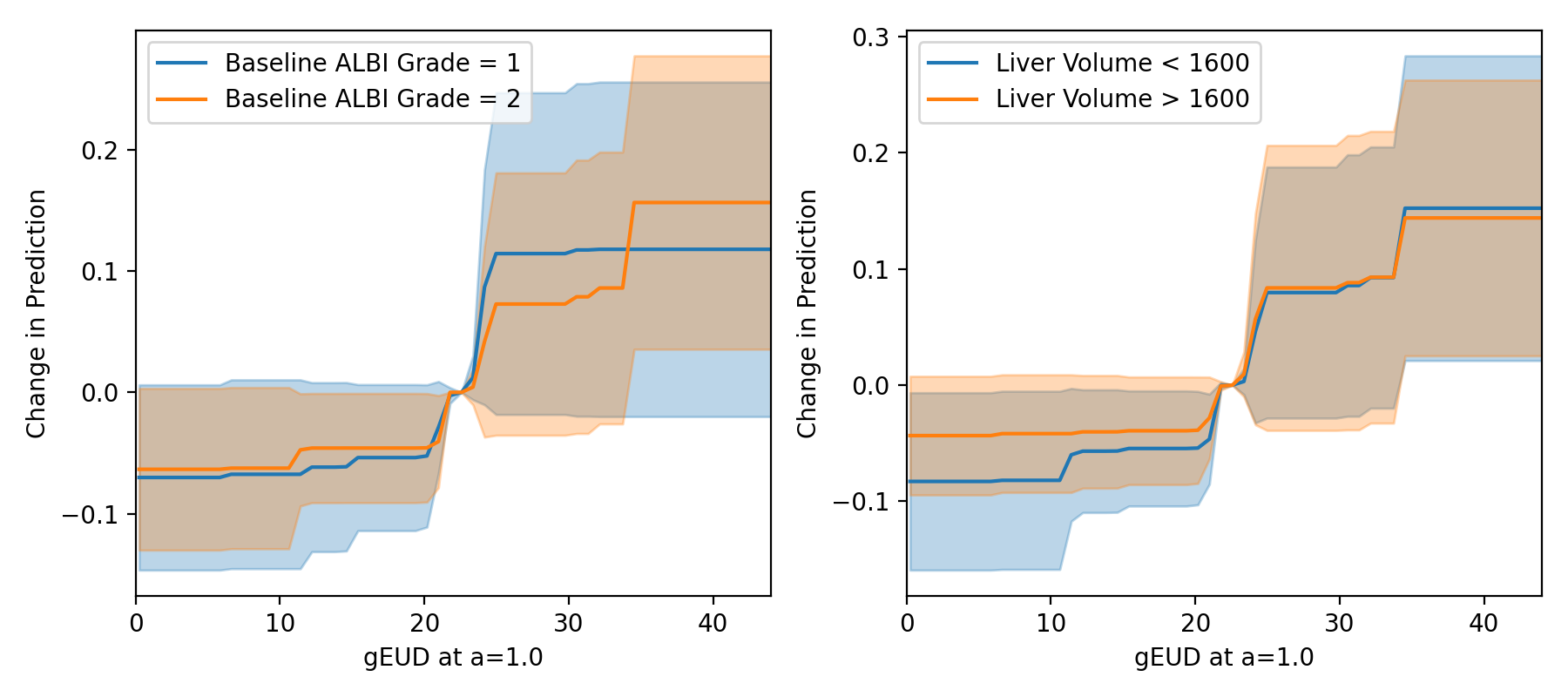


**Supplementary Figure 2.** **Impact of gEUD on patient subpopulations.** The impact of the gEUD on the prediction for each subpopulation was estimated empirically by altering its input value and recording the corresponding change in model’s output. Red trend lines show the average relative change in prediction. Blue shaded areas indicate the plus/minus one standard deviation range. A) Impacts of the gEUD on patients with a baseline ALBI grade of 1 or 2. B) Impacts of the gEUD on patients with a liver volume smaller or larger than 1,600 cm^3^.


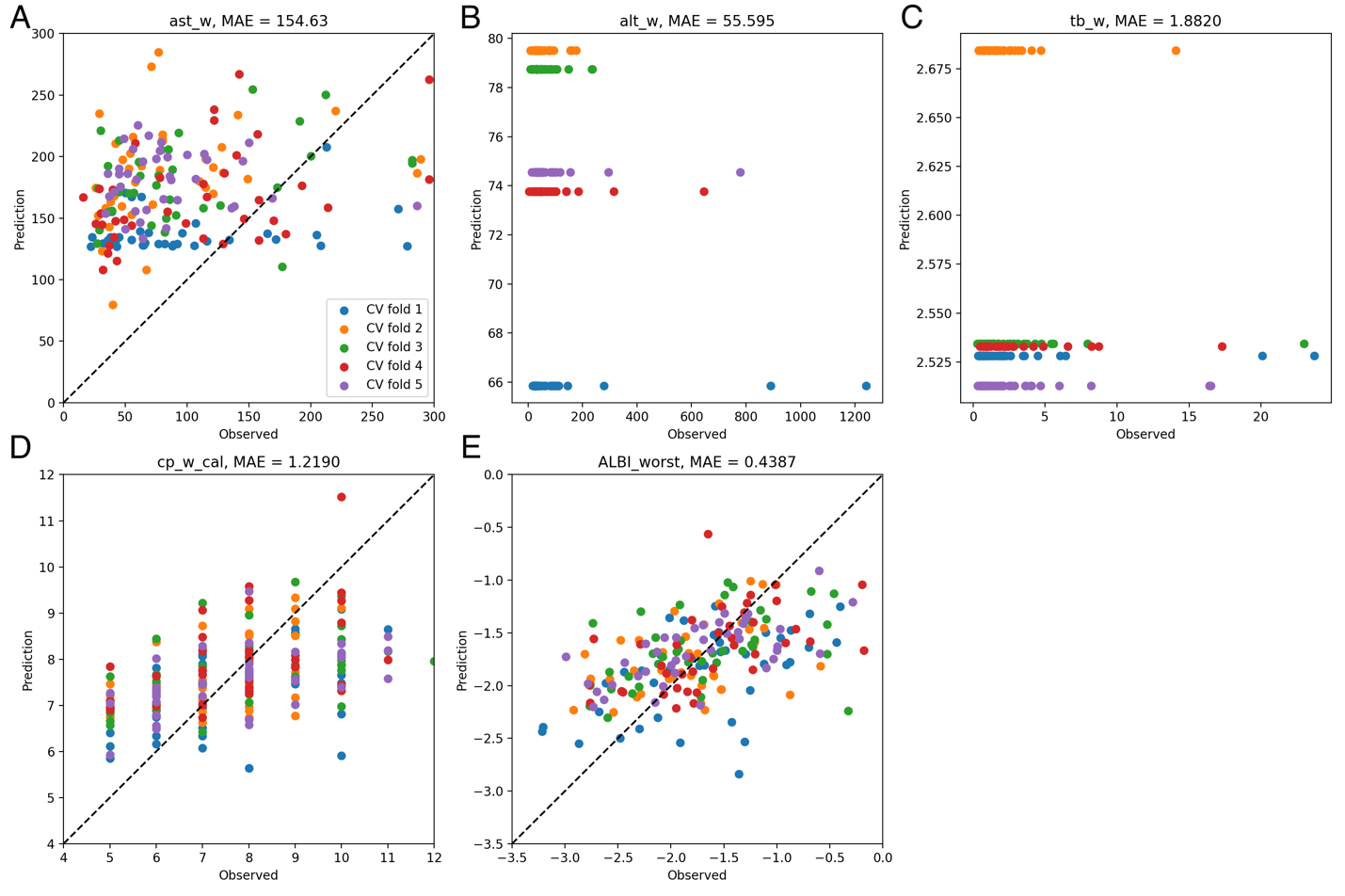


**Supplementary Figure 3.** **Predictability of the raw outcome scores using penalized linear regression.** Ridge and LASSO models were evaluated, and all input features were used. Scatter plot comparing the observed versus the predicted raw outcome scores. Results from each 5-fold cross-validation is shown with distinct color. Black dashed lines indicate the perfect prediction. Mean absolute errors are shown in the plot title. A) Worst AST. B) Worst ALT. C) Worst total bilirubin. D) Worst Child-Pugh score. E) Worst ALBI score.
